# Dr. L. W. Carpenter

**Published:** 1915-01

**Authors:** 


**Dr. L. W. Carpenter,** Cleveland Homoeopathic 1866, died at his home in Trumansburg, November 8, 1914, aged 81.

